# Mechanisms underlying seizures and hypothermia during busulphan administration

**DOI:** 10.1038/s41409-025-02608-z

**Published:** 2025-05-03

**Authors:** Ibrahim El-Serafi, Sofia Berglund, Fadwa BenKessou, Alina Codita, Maryam Saghafian, David Lindskog, Matthijs C. Dorst, Gilad Silberberg, Manuchehr Abedi-Valugerdi, Wenyi Zheng, Rui He, Manon Renault, Weiying Zhou, Chao Yu, Massoud Vosough, Sandra Oerther, Ying Zhao, Jonas Mattsson, Moustapha Hassan

**Affiliations:** 1 Experimental Cancer Medicine (ECM), Department of Laboratory Medicine, Karolinska Institutet, Stockholm, Sweden; 2 Basic Sciences Department, College of Medicine, Ajman University, Ajman, UAE; 3 Clinical Research Center and Center of Allogeneic Stem Cell Transplantation (CAST), Karolinska University Hospital Huddinge, Stockholm, Sweden; 4 Department of Oncology-Pathology, Karolinska Institutet, Stockholm, Sweden; 5 Department of Neurobiology, Care Sciences and Society, Division for Neurogeriatrics, Karolinska Institute, Stockholm, Sweden; 6 Department of Neuroscience, Karolinska Institutet, Stockholm, Sweden; 7 Department of Pharmacology, College of Pharmacy, Chongqing Medical University, Chongqing, China; Chongqing Key Laboratory of Drug Metabolism, Chongqing, China, Key Laboratory for Biochemistry and Molecular Pharmacology of Chongqing, Chongqing, China; 8 Chongqing Key Laboratory for Pharmaceutical Metabolism Research, College of Pharmacy, Chongqing Medical University, Chongqing, 400010 China; 9 Department of Regenerative Medicine, Cell Science Research Center, Royan Institute for stem Cell Biology and Technology, Academic Center for Education, Culture and Research (ACECR), Tehran, Iran; 10 Gloria and Seymour Epstein Chair in Cell Therapy and Transplantation, Princess Margaret Cancer Centre and University of Toronto; Princess Margaret Cancer Centre, University Health Network, Toronto, ON Canada

**Keywords:** Chemotherapy, Haematopoietic stem cells, Translational research

## Abstract

Busulphan (Bu) is used as a part of the conditioning regimen prior to HSCT. Neurotoxicity is one of Bu major adverse-effects. We investigated the kinetics of busulphan and its metabolites (tetrahydothiophene, tetrahydrothiophene-1-oxide, sulfolane, 3-OH-sulfolane) in patients and mice as well as the mechanisms underlying CNS-toxicity in mice. Busulphan metabolites were detectable in plasma and urine up to 72-h after the last Bu-dose. Sulfolane levels were high and reached maximum concentration at the time-point reported for the convulsions’ occurrence. Mice were treated with either busulphan or one of its metabolites, separately. Sulfolane treated-mice showed the highest brain exposure (AUC_brain_/AUC_plasma_). Seizures and hypothermia were observed after sulfolane administration, accompanied with a significant decrease in calbindin-28k concentrations in the brain. Behavior changes but no signs of convulsions were seen in mice treated with lower sulfolane doses. Moreover, a reduction of spontaneous events during whole-cell patch clamp recordings from pyramidal neurons was observed following bath application of sulfolane. In conclusion, these are the first results showing that sulfolane is the major cause of seizures and hypothermia. Sulfolane concentration in plasma mirrors its concentration in the brain. The role of calbindin-D28K in CNS-toxicity and susceptibility to future neurodegenerative diseases should be investigated.

## Introduction

Hematopoietic stem cell transplantation (HSCT) is a curative treatment for several hematological and solid malignancies. The alkylating agent busulphan (Bu) is used in high doses as a part of conditioning prior to HSCT. Busulphan is metabolized mainly in the liver through conjugation with glutathione (GSH) via glutathione transferase A1 (GSTA1) [1]. The conjugation produces an unstable sulfonium ion that is rapidly degraded to tetrahydrothiophene (THT) [2]. THT is reabsorbed from bile to be oxidized to tetrahydrothiophene 1-oxide (THT 1-oxide), which is further oxidized to sulfolane (Tetrahydrothiophene 1,1-dioxide), that is finally oxidized to 3-hydroxysulfolane (3-OH sulfolane) [2, 3]. We and others have reported the involvement of flavin-containing monooxygenase 3 (FMO3) and cytochrome P450 (CYPs) Bu metabolic pathway via the oxidation of THT and probably THT 1-oxide that can affect Bu kinetics [4, 5].

Studies in monkeys and man have shown that Bu can easily cross the blood-brain barrier (BBB) [3, 6, 7]. Only 1–2% of Bu is excreted in the urine unchanged, while about 70% of the dose is excreted in rats urine within 72 h as 3-OH-sulfolane (39%), THT 1-oxide (20%), and sulfolane (13%) [2, 6].

Several old occupational studies have reported the neurotoxicity of sulfolane [8, 9]. Rats treated with an intraperitoneal (i.p.) injection of sulfolane are less active than those receiving saline [8]. Other studies have reported that sulfolane induced convulsions and hypothermia in animals [8–10].

Previous studies reported that about 10% of patients receiving high doses of Bu experience seizures [11, 12]; therefore, anticonvulsant prophylaxis such as phenytoin or diazepam [11, 13–17] are included in the conditioning regimen. However, the mechanisms underlying these seizures are not fully understood.

In the present investigation, we studied the kinetics of Bu and its metabolites in patient’s plasma and urine and in mouse plasma and organs. We further investigated the effect of Bu and its metabolites on mouse behavior, neurotoxicity markers, and changes in body temperature in order to understand the mechanism underlying the CNS-toxicity.

## Materials and methods

Material used in the pharmacokinetics and ELSIA studies are listed in the complementary data.

### Patients

Patients receiving Bu prior to HSCT (*n* = 18) from the Center for Allogeneic Stem Cell Transplantation (CAST) at Karolinska University Hospital-Huddinge-Sweden were recruited to the study. Patients’ characteristics are listed in Table 1. The study was approved by the Regional Ethics Committee (DNR 425/97, 619-17) in accordance with the Helsinki Declaration, and informed consent was obtained from all patients or their guardians. The sample size was calculated considering a 10% margin of error and a 95% confidence interval.

**Table 1:** Patients’ characteristics.

Patients characteristics	
Median patient age	33.5 (12–66)
Number of children	1
Number of adults	17
Females	9
Males	9
Diagnoses	
ALD	1
AML	15
CML	2
Conditioning regimen	
Bu + Cy	18

*ALD* adrenoleukodystrophy, *AML* acute myeloid leukemia, *CML* chronic myelogenous leukemia, *Bu* busulphan, *Cy* cyclophosphamide.

Patients received oral Bu (b.i.d., 2 mg/kg/day for 4 days) [18]. Blood samples were collected in EDTA vacutainer tubes for therapeutic drug monitoring (TDM) and dose adjustment. Extra samples were collected at 24, 48, and 72 h after Bu last-dose. Plasma was separated by centrifugation (3000 *g*, 5 min) and stored at −20 °C. Urine samples were collected from 11 patients regularly and stored at −20 °C.

Bu concentrations were measured by gas chromatography with an electron capture detector (GC-ECD) (SCION 436-GC; Bruker) [19], while Bu four metabolites, were measured using gas chromatography-mass spectrometry (GC-MS) (Agilent 6890N series GC equipped with Agilent 7683 auto-injector and Agilent 5973N mass selective detector, Santa Clara, CA, USA) [20].

### Mice

Male C57BL/6N mice (6–8 weeks) were purchased from Charles River (Koln, Germany). The study was approved by Stockholm South ethical committee (Dnr. S119-12 and (S53-13). Details regarding the mice housing are reported in the supplementary data.

### Pharmacokinetic study

Busulphan dose was calculated to correspond to its dose in patients prior to HSCT and its equimolar dose for the metabolites. Mice received single doses (i.p) of Bu, THT, or THT-1-oxide at doses of 25, 8.8, and 10.4 mg/kg, respectively. All three compounds were dissolved in DMSO and diluted using NaCl (9 mg/mL) prior to administration (200 µL, 5% DMSO). Sulfolane and 3-OH sulfolane were dissolved in phosphate-buffered saline (PBS) and administered separately i.p. at doses of 12 and 13.6 mg/kg, respectively (200 µL). No blinding was carried out.

Blood was collected by cardiac puncture at different time-points for the kinetics of Bu and its metabolites (*N* = 3 per time point). Plasma was separated and stored at −20 °C. After blood collection, mice were euthanized, perfused, then organs were collected and snap-frozen.

Prior to homogenization, frozen organs were thawed at room temperature, and PBS was added (4 and 3 mL/g for liver/kidney and brain samples, respectively). The homogenates were centrifuged at 6000 × *g* for 18 min, and the supernatant was collected. Bu and its metabolites were quantified in organ homogenates and plasma using GC-MS [20].

### Behavioral and neurological studies

Mice were transported from their room to a behavioral lab within the same facility and were divided into 5 groups. Each group (*n* = 4) was injected (i.p.) with only one of the compounds. Mice from one cage were subsequently placed in an MIII empty cage containing wood chips and a red house as a shelter. Mice were closely monitored from a distance of less than 1 m for one to two hours. The same experienced observer performed consecutive observations of each mouse for 1 min, spaced at 10–15-min intervals.

The same experiment was repeated to video-record any changes in mice for 1 h. Temperature changes were regularly recorded using a thermos camera. In seizure occurrence, mice were sacrificed when the episode exceeded 10 s.

Brains were collected, stored, and prepared as previously described. The supernatants were measured by ELISA for different neurotransmitters: dopamine, 5-hydroxytryptamine (5-HT), glutamate, GABA, and calbindin-28k. An extra group of mice, receiving Bu i.p. once daily for four days, was added to this experiment. Two control groups were run in parallel. One group received DMSO (5%), while the other received PBS in total volume of 200 µL.

### Ex vivo electrophysiology

Slice preparation and whole-cell patch clamp are mentioned in details in the supplementary data. The following compounds were applied during ex vivo electrophysiology: Bu (100 µM) was prepared as 100 mM stock in DMSO and diluted in oxygenated ACSF. Sulfolane (1 mM) and THT 1-Oxide (2 mM) were diluted directly in oxygenated ACSF to the appropriate concentrations. Other compounds were not tested since they showed no significant effect in the previous experiments.

Responses to various current pulses were analyzed using custom scripts written for Igor Pro-6.3. Spontaneous events were detected by examining the smoothed wave’s first- and second derivatives. The minimum detection threshold for post-synaptic events was set to 6 standard deviations above the baseline. Membrane time constants were determined from a brief (5 ms) hyperpolarizing current pulse.

### Data analysis

Enhanced ChemStation software G1401BA version E.02.02.1431 (Agilent, Santa Clara, CA, USA) was utilized for the collection of chromatograms and quantitative data, while kinetics estimations were performed using WinNonLin software (standard edition, version 2.0). All statistical analyses and graphs were performed using GraphPad Prism (version 4.0, GraphPad Software, Inc.). Values were considered significant when the *P* value < 0.05.

## Results

### Patients

#### Pharmacokinetics in plasma

**Fig. 1:**
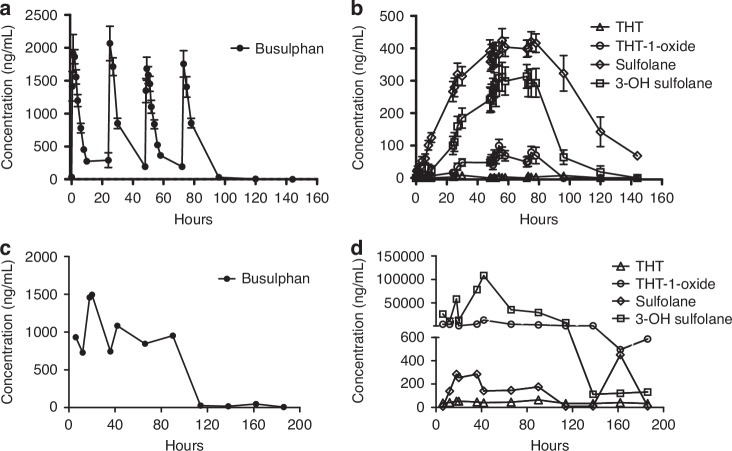
The concentrations of busulphan and its four metabolites in plasma and urine of patients during the period of conditioning with Bu and up to 3 days after the last dose administration. **a** Bu concentrations in plasma. **b** The 4 metabolites concentrations in plasma. **c** Bu concentrations in urine. **d** The 4 metabolites concentrations in urine. Mean ± standard error of the mean (SEM).

Patients mean AUC/dose over the 4-day conditioning was 11542 ± 452 ng.h/mL (2815.1 ± 110.2 μmol.min/L). Busulphan maximum concentrations were detected 1–2 h post-administration, and it was undetectable 24 h after Bu last dose (Fig. 1a). THT concentrations were hardly detectable, indicating that THT is rapidly oxidized to THT 1-oxide. THT 1-oxide started to emerge in plasma after the second day of conditioning to reach a maximum concentration of 99 ± 30 ng/mL (0.95 ± 0.3 μM) 6 h after Bu 5^th^ dose. Sulfolane plasma concentrations were the highest among the metabolites as it detectable 1 h after Bu 1^st^ dose and reached maximum plasma levels (424 ± 159 ng/mL, 3.5 ± 1.3 μM) 8 h after the 5^th^ dose. Sulfolane reached a steady-state level that maintained throughout the Bu- and was detectable 60 h post Bu last dose (68.5 ± 13 ng/mL, 0.6 ± 0.1 μM). 3-OH sulfolane had a similar pattern as sulfolane; however, the plasma levels were lower. 3-OH sulfolane was emerging in plasma after Bu 1^st^ dose; the maximum concentrations were reached 6–8 h after the 5^th^ dose (mean concentration 313 ± 150 ng/mL, 2.3 ± 1.1 μM) and it was detectable 24 h after Bu last dose (Fig. 1b).

#### Pharmacokinetics in urine

Bu concentrations were constantly high upon the start of the conditioning. The highest concentrations were detected 12 h after the first dose (1.5 µg/mL, 6.1 μM). Bu was detectable in urine up-to 3 days post Bu last dose (Fig. 1c). THT and sulfolane urine levels were relatively low in all samples (mean concentration: 47.3 ng/mL [0.5 μM] and 145.3 ng/mL [1.2 μM], respectively). THT 1-oxide was high in urine, particularly in samples taken 4–6 h post-dose (mean maximal concentration 5.9 µg/mL, 56.6 μM). The highest concentrations of Bu-metabolites were detected for 3-OH sulfolane (mean maximal concentration: 35.1 µg/mL, 257.8 μM 4–6 h post-dose) (Fig. 1d).

### Mice

#### Pharmacokinetics in plasma

**Fig. 2:**
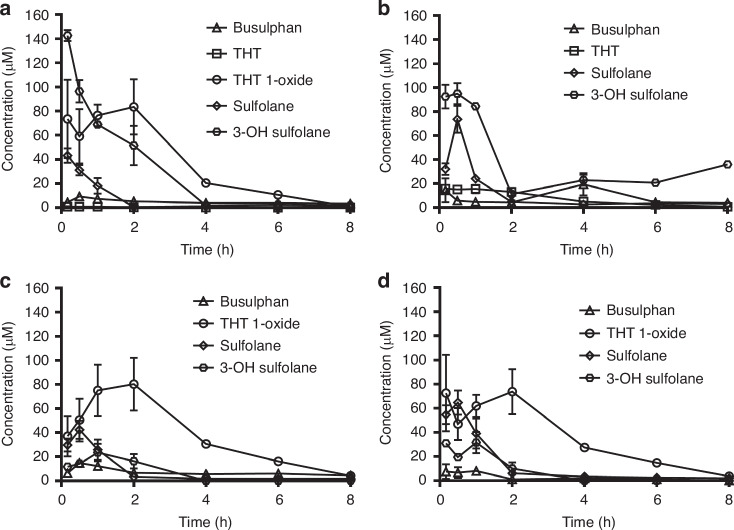
The concentrations busulphan and its metabolites in mice 10 min - 8 h post-injection. **a** plasma, (**b**) liver, (**c**) brain and (**d**) kidneys; Mean ± SEM.

Both Bu and THT levels were low (<8 μM and <0.46 μM respectively) throughout all timepoints. THT 1-oxide peak plasma concentration was 83.6 μM, measured 2 h after injection. Sulfolane peaked at 43.3 μM 10 min post-injection and was completely eliminated from plasma 6 h post-administration. Finally, 3-OH sulfolane peak plasma concentration was 142.8 μM after 10 min (Fig. 2a). Accordingly, the largest calculated maximum concentration (C_max_) was found for 3-OH sulfolane followed by THT-1-oxide. Busulphan showed the longest elimination half-life followed by THT and THT-1 oxide. The shortest half-life was observed for sulfolane. THT-1-oxide had the highest AUC followed by 3-OH sulfolane (Table 2).

**Table 2:** Pharmacokinetic parameters for Bu and its metabolites in mice plasma and organs; Mean ± SD.

Compounds	AUC [μmol.min/L]	HL [h]	C_max_ [μmol/L]
	Plasma		
Bu	3329.4 ± 521.4	4.48 ± 0.96	7.67 ± 0.51
THT	112.2 ± 5.4	3.19 ± 0.23	0.34 ± 0.01
THT 1-oxide	17405.4 ± 5548.2	1.98 ± 0.86	92.10 ± 13.47
Sulfolane	2707.8 ± 208.8	0.57 ± 0.07	54.45 ± 3.38
3-OH sulfolane	12550.2 ± 3011.4	1.18 ± 0.36	100.68 ± 39.20
	Liver		
Bu	8826 ± 3820.8	19.36 ± 9.35	5.18 ± 0.31
THT	3955.8 ± 548.4	2.41 ± 0.45	17.24 ± 1.11
THT-1-Oxide	–	–	–
Sulfolane	6010.2 ± 264.6	0.32 ± 0.04	217.38 ± 29.79
3-OH Sulfolane	112035.6 ± 196254	4.84 ± 186.99	94.73 ± 4.95
	Brain		
Bu	4579.8 ± 1366.2	3.85 ± 1.58	12.21 ± 1.64
THT	–	–	–
THT-1-Oxide	12539.4 ± 2170.8	1.17 ± 1.01	81.54 ± 5.61
Sulfolane	3925.8 ± 1255.8	1.06 ± 0.46	42.84 ± 8.43
3-OH Sulfolane	3295.8 ± 709.8	0.65 ± 32.74	21.63 ± 2.34
	Kidneys		
Bu	1039.8 ± 352.8	1.22 ± 0.62	8.26 ± 1.31
THT	–	–	–
THT-1-Oxide	16959.6 ± 8016.6	2.12 ± 1.39	80.45 ± 17.97
Sulfolane	6063 ± 1397.4	0.96 ± 0.30	73.20 ± 10.79
3-OH Sulfolane	3197.4 ± 832.8	1.11 ± 0.44	29.29 ± 3.44

*AUC* area under the curve, *Bu* busulphan, *Cmax* maximum plasma concentration, *HL* half-life, *THT* tetrahydrothiophene.

#### Pharmacokinetics in organs

The concentrations of Bu and its metabolites in mice livers, brains, and kidneys were measured at different time points (Fig. 2b–d, respectively, Table 2). Busulphan levels were low throughout all organs and time points. THT was detected only in the liver at low concentrations. THT-1-oxide reached peak organ concentrations at 2 h for the brain and kidneys. The mean concentration of THT-1-oxide for the brain and kidneys were 80.39 μM and 73.78 μM, respectively. Mice injected with sulfolane displayed maximum concentration peaks 30 min post-injection for all organs. The highest mean concentration of sulfolane was 73.6 μM in the liver. The highest concentration of the metabolites in the liver was 3-OH sulfolane with a mean concentration of 92.6 μM 30 min post-injection.

The largest AUC calculated was for 3-OH sulfolane in liver. In brain and kidney, the largest AUC calculated was for THT-1-oxide. Busulphan had the highest half-life in the liver and brain, while THT-1-oxide had the highest half-life in the kidneys. C_max_ varied among organs, with the highest value being sulfolane in the liver, while THT-1-oxide had the highest C_max_ for both brain and kidney tissues.

An important factor in assessing neurotoxicity was the distribution of Bu and its metabolites in mouse brains and plasma. Bu and sulfolane displayed the highest AUC_brain_/AUC_plasma_ ratio (Table 3).

**Table 3:** Distributions between brain and plasma for busulphan and its metabolites.

Compound	Ratio AUC _Brain_/AUC _Plasma_
Bu	1.38
THT	–
THT-1-Oxide	1.07
Sulfolane	1.45
3-OH Sulfolane	0.26

*AUC* area under the curve, *Bu* busulphan, *THT* tetrahydrothiophene.

### Behavioral and neurological changes

Mice injected with Bu, THT and sulfolane showed several behavioral changes that started 10 10 min post-injection. These changes included digging, rearing, burying their head into bedding, nibbling at pieces of bedding, licking, grooming, pushing bedding and sniffing. All mice were awake in the respective corners/nests 1–1.5 h post-injection. No significant observations were recorded when mice were injected with THT-1-oxide, 3-OH sulfolane, or in both control groups. Detailed behavioral changes are listed in the supplementary data.

Myoclonic **s**eizures were observed in 40% of the mice injected with sulfolane 20–30 min post-injection (Supplementary Video 1). One mouse injected with Bu had myoclonic seizures (partial attack) after 45 min.

**Fig. 3:**
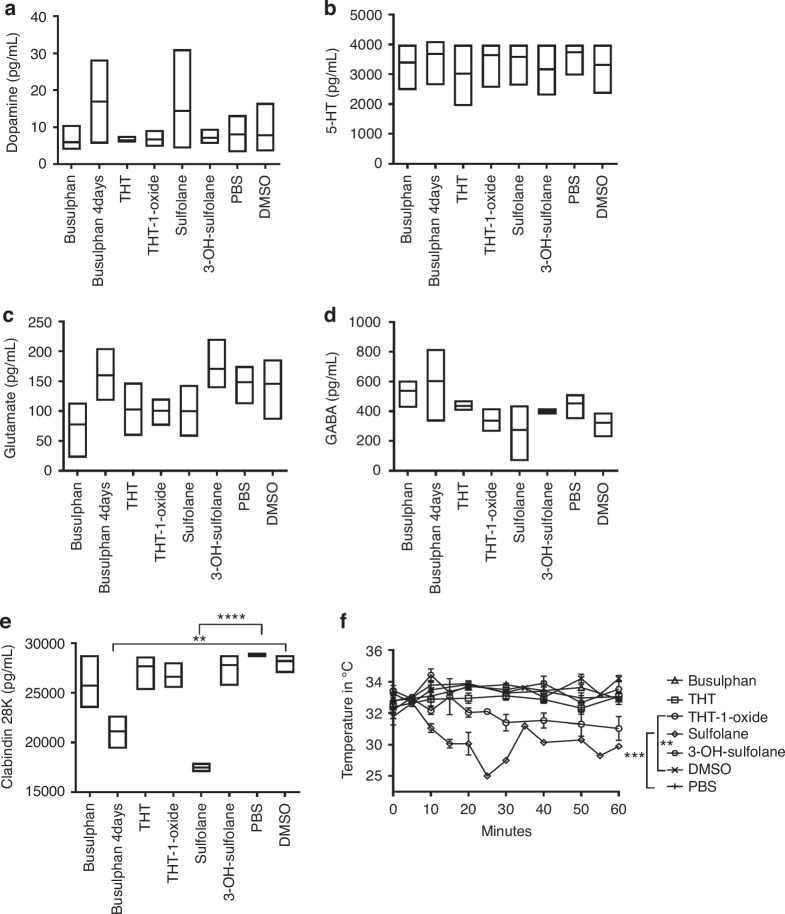
Changes of neurotransmitters and temperature in mice. Neurotransmitters were measured in mouse brain after i.p injection of Bu or one of its metabolites: **a** Dopamine, **b** 5-hydroxytryptamine (5-HT), **c** Glutamate, **d** GABA and **e** Calbindin 28 K. Temperatures were measured in mice at several time points after i.p injection of Bu or one of its metabolites; Mean ± SEM (**f**).

In sulfolane-treated mice, a significant (*P* < 0.0001) decrease in calbindin-28k concentrations was observed in the brains compared to the control group. Calbindin-28k was also significantly decreased after Bu-administration for 4-days. No other significant changes were observed (Fig. 3a–e). Hypothermia was significant (*P* < 0.005) in mice injected with sulfolane, as well as THT-1-oxide, compared to the control group (Fig. 3f).

### Ex vivo electrophysiology

**Fig. 4:**
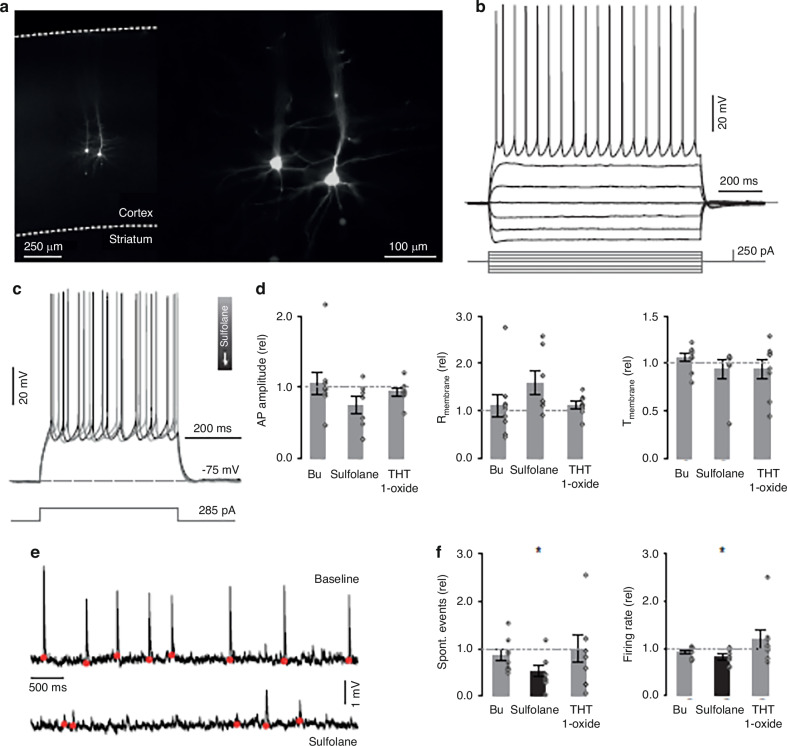
Effects of busulphan, sulfolane, and THT-1-oxide application on cortical neurons. **a** Location and morphology of stained pyramidal neurons that were visualized post-hoc. **b** Electrophysiological parameters such as I-V relationships for each pyramidal neuron were recorded pre- and post-compound application. **c** The response to suprathreshold depolarizing current steps was recorded during application. **d** Action potential amplitude, membrane resistance, and membrane time constant of pyramidal neurons relative to baseline, following bath application of Bu, sulfolane, and THT-1-Oxide. **e** spontaneous input on a pyramidal neuron prior to and following the application of sulfolane. **f** The maximum firing rate in pyramidal neurons decreased following the application of sulfolane. Spontaneous events decreased in frequency following the application of sulfolane but not Bu or THT-1-Oxide. Error bars indicate ±SEM.

Whole-cell patch clamp recordings from pyramidal neurons in layer 5 of the somatomotor cortex were performed. Pyramidal neuron identity was confirmed by their typical I-V response (Fig. 4a) and characteristic morphology following post-hoc immunohistochemistry staining (Fig. 4b). A range of current steps and ramps was injected to probe the intrinsic electrophysiological parameters of each neuron prior to and following bath application of Bu, sulfolane and THT 1-oxide. During wash-in, a brief depolarizing current pulse was applied to monitor potential gradual changes in electrophysiological parameters (Fig. 4c).

No changes in action potential amplitude, resting membrane resistance, or membrane time constant were observed following the bath application of these compounds (Fig. 4d). Only following bath application of sulfolane, a decrease in spontaneous post-synaptic events (*n* = 8, *P* = 0.036, Z = 2.1, Wilcoxon signed ranks test) (Fig. 4e, f) as well as a in maximum pyramidal neurons firing rate (*n* = 7, *P* = 0.028, Wilcoxon signed ranks test) (Fig. 4f) were observed. Neurons exposed to sulfolane exhibited altered intrinsic properties and a change in external input. This suggests sulfolane enacts physiologically observable changes in neural circuits that are exposed to this compound.

## Discussion

Busulphan administration prior to HSCT is associated with various adverse effects, including seizures [21–26]. Approximately 10% of the patients without seizure prophylaxis experienced seizures that mostly occur on the second day (after 6–7 doses when Bu is administered q.i.d) [27, 28]. Convulsions were also reported to occur 18–24 h after Bu last dose [29], indicating that Bu itself might not be the cause of seizures due to its short half-life.

Historically, sulfolane, as an industrial product, was reported to induce neurotoxicity in animals, including convulsions and hypothermia [8–10, 30]. However, there are no reports regarding its toxic effect as a Bu-metabolite. Additionally, no underlying mechanism has been reported.

In the current investigation, sulfolane showed the highest concentrations in patients’ plasma, reaching the peak at the time previously reported for seizures. Bu metabolites were detectable in plasma and urine up to 72 h post last-dose. In mice, sulfolane displayed the highest AUC_brain_/AUC_plasma_. Seizures and hypothermia were observed after sulfolane injection with a significant decrease in calbindin-28k concentrations in brain. Moreover, a decrease in spontaneous events during whole-cell patch clamp recordings from pyramidal neurons was observed following bath application of sulfolane.

Sulfolane appeared early in patients’ plasma (1 h after 1^st^ Bu-dose). In contrast to Bu, the plasma concentrations of sulfolane increased steadily and reached maximal steady-state levels around 6–8 h after the 5^th^ dose Bu administration, which is in the same time frame for the previously reported convulsions in patients [27, 28]. Moreover, sulfolane was detected in patients’ plasma up to 60 h after Bu last dose, which can also be correlated to the late convulsions previously reported [29]. In agreement with our findings, sulfolane, in addition to other Bu metabolites, was detected in pre-graft plasma from allogeneic HCT recipients [31].

Accordingly, sulfolane quantification can be valuable in patients, especially those at high risk for convulsions. Patients with high sulfolane levels are at high risk for seizures and those are the ones who need prophylactic treatment. In this case, there will be no need for prophylaxis administration for all patients or, at least, it can be provided as a shorter course (personalized prophylaxis). This will certainly reduce the treatment load, potential drug-drug interaction, seizure prophylaxis adverse effects in addition to the economic impact.

However, further investigation in patients was not possible, so we performed animal experiments to confirm our findings and explore the underlying mechanisms.

In mice, both THT-1-oxide and sulfolane concentrations were high in plasma and organs at early time points with relatively high AUC in both plasma and organs. Additionally, sulfolane had the highest AUC_brain_/AUC_plasma_ ratio, which might explain the convulsions’ episodes reported in animals and seen in patients.

During the behavior study, an overall decrease in movement was observed in mice injected with sulfolane during the open field tests, and burrowing was significantly decreased. These results coincide with a previous study, where neurotoxicity was observed in mice treated with sulfolane in the form of hypoactivity and convulsions [10]. Sulfolane reaches a peak concentration in the brain at the same time we observed both seizures and hypothermia (30 min), which is in agreement with the previous reports [8–10, 30]. In addition to sulfolane, THT-1-oxide had high concentrations and AUC in brain and induced hypothermia.

We and others have previously reported the involvement of CYPs in the Bu metabolic pathway [4, 5]. CYPs are expressed in the brain which can induce THT-1-oxide oxidation to sulfolane that can increase sulfolane concentrations in the brain and hence, more neurotoxicity [32, 33].

Mice injected with sulfolane had significant decrease in calbindin-28k in brain. Calbindin-28k is a major calcium-binding protein that is vital in maintaining calcium homeostasis and its decrease was previously reported to induce convulsions [34]. Interestingly, mice injected with 4-d Bu also showed a significant reduction in calbindin-28k levels which strongly suggests that sulfolane is the main cause of convulsions and indeed, several Bu-doses are required to reach steady state concentrations and induce its effect. Long-term decrease in Calbindin-28k was reported to be associated with CNS toxicity and increased susceptibility to neurodegenerative diseases. Given its crucial neuroprotective role, Calbindin-28k may serve as a biomarker for long-term neurodegeneration in patients who went HSCT as children [35, 36].

On the other hand, hypothermia can alter calcium accumulation and handling [37, 38], as well as, calbindin-28k concentrations [39]. However, further investigations are required to explore this correlation and the underlying mechanisms between calbindin-28k, convulsions, and hypothermia.

Finally, the whole-cell patch clamp test has explained the neurotoxic effect of sulfolane. Exposure to sulfolane has decreased spontaneous post-synaptic events, reduced the maximum firing rate for pyramidal neurons, and altered the neurons both intrinsic properties and external input.

From another clinical perspective, we have noticed the accumulation of Bu metabolites for several days in patients’ plasma and urine after Bu last dose. Busulphan/cyclophosphamide (Cy) are commonly used in the clinical setting for conditioning prior to HSCT, as Cy is given to the patients 24 h after the end of Bu treatment. Cyclophosphamide is a prodrug that is bioactivated via CYP2B6 and several other CYPs [40–42]. Since CYPs are also involved in the Bu metabolism [4, 5], the presence of Bu metabolites in patients’ blood during Cy treatment can alter Cy bioactivation and increase its toxicity. Additionally, the 24 h interval between Bu and Cy was reported to decrease the incidence of hepatotoxicity [43, 44] while alteration of the administration order to Cy-Bu gave the same outcome but reduced adverse effects [45–48]. Cy bioactivation is a rate-limited step that is controlled mainly by cytochrome P450 oxidoreductase (POR). It is well-known that this bioactivation is rather fast for Cy and its active metabolite (4-OH-Cy) half-life in biological fluids is 4 min only [49]. Giving Cy upfront will give the CYPs the chance to recover before Bu administration and should not alter its metabolism. Accordingly, the Bu/Cy conditioning protocols should be revised.

In conclusion, this is the first study presenting the kinetic of busulphan and its metabolites in patients’ plasma as well as in mice organs and identifying sulfolane as the main cause of neurotoxicity. Monitoring sulfolane kinetics is clinically essential to minimize its neurotoxicity considering the long-term effect of Calbindin-28k decrease. Moreover, the accumulation of the metabolites in plasma after Bu last dose might alter the kinetics of the subsequently administered drugs, which should be considered in order to increase the treatment efficacy and improve the clinical outcome.

## Supplementary information

Supp. video 1

Supplementary Data

## Data Availability

The datasets generated during and/or analyzed during the current study are available from the corresponding author upon request.
