# Supplementary material for: Mechanisms underlying seizures and hypothermia during busulphan administration
Source: Bone Marrow Transplant. 2025 May 3;60(8):1120–8. doi: 10.1038/s41409-025-02608-z (PMC12321567; doi:10.1038/s41409-025-02608-z)
Supplement: Supplementary file 2 — Supplementary Data [file 41409_2025_2608_MOESM2_ESM.docx]

**Mechanisms Underlying Seizures and Hypothermia During Busulphan Administration**

**Supplementary Data**

**Materials and Methods**

**Materials**

The compounds were purchased with a purity (>96%): Bu and 3 OH sulfolane (Sigma–Aldrich, Steinheim, Germany), THT and sulfolane (Sigma–Aldrich, St. Louis, USA) and THT 1-oxide (Sigma–Aldrich, Tokyo, Japan).

Neurotransmitters were measured by ELISA in mouse brain supernatants using the following kits: dopamine (Cusabio), 5-hydroxytryptamine (5-HT. Cusabio), glutamate (BlueGene), GABA (Fine Test), and calbindin-28k (Fine Test).

**Mice**

The mice were maintained according to local housing procedures in open cages or individually ventilated cages (IVCs) under pathogen-free conditions with controlled humidity (55% ± 5%), 12 hours light/dark, temperature (21^○^C ± 2^○^C), and HEPA-filtered air. Cages had bedding material and enrichment in the form of red house or nesting material and burrows. Mice had access to irradiated mouse standard chow and water *ad libitum.*

The mice could acclimatize without human intervention, except for husbandry procedures, for at least two weeks following arrival in the laboratory.

***Ex vivo* electrophysiology**

1. **Slice preparation**

Adolescent mice aged P39 to P50 were anesthetized with isoflurane (VM Pharma AB, Sweden) prior to being decapitated, whereupon brains were extracted while submerged in an ice-cold cutting solution consisting of (in mM) KCl 2.5, NaH_2_PO_4_ 1.25, CaCl_2_ 0.5, MgCl_2_ 7.5, Glucose 10, NaHCO_3_ 25, and Sucrose 205. Parasagittal sections of 250 μm thickness were cut using a VT1200S Vibratome (Leica, Japan) at an angle of 10° and subsequently left to recover for 30 minutes in 35°C artificial cerebrospinal fluid (ACSF) containing (in mM): NaCl 125, KCl 2.5, MgCl2 1, NaH2PO4 1.25, CaCl2, Glucose 25, and NaHCO3 25. Slices were maintained at room temperature from recovery until recording at 35°C. Cutting solution and ACSF were continuously infused with carbogen (95% O2, 5% CO2) throughout the procedure.

1. **Whole-cell patch clamp**

Borosilicate glass pipettes were pulled using a P1000 Micropipette Puller (Sutter Instrument, U.S.A.) for a 6 – 8 MΩ resistance and filled with intracellular solution. A high-chloride intracellular solution was used containing (in mM): K-gluconate 105, KCl 30, Na_2_-Phosphocreatine 10, HEPES 10, ATP-Mg 4, GTP-Na 0.3. Under these conditions, chloride-based post-synaptic events can appear excitatory. For post-hoc staining experiments, 0.1% neurobiotin was added to the intracellular solution. Neurons were selected by Infrared-Differential Interference Contrast (IR-DIC) imaging on a BX51WI (Olympus, Japan) upright microscope using a 40x long-working-distance immersion objective. Pyramidal neurons were identified by their large somata, and their identity was confirmed electrophysiologically in whole-cell patch clamp configuration. Up to three pyramidal neurons were recorded simultaneously. Once a whole-cell patch was achieved, voltage responses were recorded at VH = -75 mV in current-clamp mode on a MultiClamp 700B (Molecular Devices, U.S.A.), digitized at 10 KHz on an ITC-18 (HEKA, U.S.A.) and acquired with Igor Pro 6.3 (Wavemetrics, U.S.A) through the PulseQ package. Baseline electrophysiological parameters were recorded without drugs present in the ACSF. During compound application, a depolarizing current pulse was provided every 30 seconds to test for gradual physiological changes and monitor access resistance. Compounds were applied through the bath perfusion system and perfused for at least 15 minutes prior to recording.

**Results**

**Mice**

1. **Behavioral and Neurological changes**

**Mice injected with Bu:** In the first 10 minutes after the injection, four out of four mice engaged in walking, digging, and rearing. Mice were active, walking, rearing, and burying their head into the bedding, nibbling at pieces of bedding material, licking, and grooming. Later (20-30 minutes), mice started pushing bedding with the nose, walking, sniffing from a still position in the corner, sniffing in the air. One mouse presented with small eyes, while another mouse had retracted ears and was not moving but smelling upwards from a still position. The mice engaged in bedding, pushing, rearing, sniffing, and SAP while walking. Finally, (50-60 minutes) two mice were active in the cage, engaging in rearing, walking and sniffing activities. The other two animals were in the corner of the cage. One mouse out of these possibly presented with signs of discomfort/pain in the form of loss of balance and orbital tightening.

The mice were recovering slowly. After one and a half hours, three mice were resting in the nest area while the last one engaged in walk-sniffing behavior, rearing, and jumping.

**Mice injected with THT:** In the first 10 minutes after injection, behaviors such as sniffing-walking, grooming, digging, and licking were observed. Subsequently (after 20 minutes), the mice became less active, with 3 out of 4 coming to the "corner/nest/protected" area where they groomed their face, licked their bodies, or sniffed in the air. At 30 minutes, mice were all spending time in the corner of the cages while nibbling at the bedding, engaging in nest building/arranging or grooming. At 40 minutes, one mouse was active, while the other 3 mice were awake in the respective corners, grooming, turning in the nest, and shortly walking or digging. Later (at 50 minutes), mice engaged in activities such as: bedding, nibbling, sniffing, grooming, licking fur, and digging.

**Mice injected with sulfolane:** In the first 10 minutes after injection, all mice sought shelter either in the corner or a previously made nest, from where they sniffed at the air. Two out of four mice adopted flattened positions. One mouse presented an extended tail position. During the 20 to 30-minute interval, the mice moved slowly and often paused their locomotive activities. Later, at 40 minutes, three out of four mice were resting in the respective corners while one mouse was walking. The four mice were resting in the respective corners 50 minutes after the injection. One hour following injection, all mice were awake in the respective corners/nests.

No significant observations were recorded when mice were injected with THT-1-oxide, 3-OH sulfolane, or both control groups.
